# Milk Fat Globule Membrane Is Associated with Lower Blood Lipid Levels in Adults: A Meta-Analysis of Randomized Controlled Trials

**DOI:** 10.3390/foods13172725

**Published:** 2024-08-28

**Authors:** Alexander P. Kanon, Sarah J. Spies, Alastair K. H. MacGibbon, Maher Fuad

**Affiliations:** Fonterra Research and Development Centre, Private Bag 11029, Dairy Farm Road, Palmerston North 4472, New Zealand; akanon@ucc.ie (A.P.K.); johannie.spies@fonterra.com (S.J.S.); alastair.macgibbon@fonterra.com (A.K.H.M.)

**Keywords:** MFGM, milk phospholipids, blood lipid, cholesterol, gangliosides, metabolic health, meta-analysis

## Abstract

Cardiovascular diseases (CVDs) are the leading cause of mortality worldwide, with dyslipidemia being a significant risk factor. This meta-analysis provides a comprehensive evaluation of the impact of bovine dairy-derived milk fat globule membrane (MFGM) supplementation on blood lipid profiles in adults. A systematic search was conducted across various databases up until March 2024, resulting in the inclusion of 6 trials with a total of 464 participants. The findings indicated that MFGM phospholipid supplementation may significantly reduce total cholesterol (TC) and low-density lipoprotein (LDL) cholesterol levels. A combined analysis of the effects on TC, LDL, and triglycerides (TG) revealed a significant overall reduction in these markers. However, no significant increase or reduction was observed on high-density lipoprotein (HDL) and TG levels. Overall, MFGM phospholipid intake may significantly decrease the level of TC and LDL, while no significant changes in TG and HDL were observed. These results suggest that MFGM supplementation could be a promising dietary intervention for improving lipid profiles in adults. Nonetheless, further research is warranted to confirm these results and to better understand the potential variability in the impact of MFGM on blood lipid levels.

## 1. Introduction

Metabolic health has increasingly become a significant cause for concern worldwide, with rising incidences of obesity, diabetes, and cardiovascular diseases (CVDs) [1]. The most important behavioral risk factors for metabolic health are an unhealthy diet, physical inactivity, tobacco use, and harmful use of alcohol [2]. The effects of these behavioral risk factors may manifest as raised blood pressure, raised blood glucose, raised blood lipids, and overweight and obesity [3]. Among these, raised blood lipids are a particularly good target for intervention, as they are directly linked to the development of atherosclerosis and subsequent cardiovascular events [4]. Managing blood lipid levels through lifestyle modifications and pharmacological interventions can significantly reduce the risk of heart attack, stroke, and other complications.

Traditionally, pharmacological interventions, such as statins and other lipid-lowering drugs, have been employed to manage abnormal blood lipid levels [5]. While these medications can be effective, they often come with a range of side effects, including muscle pain, digestive problems, and increased risk of diabetes [6,7]. An important consideration is the population with borderline elevated lipid levels, i.e., those whose lipid profiles are not yet at a level warranting statin therapy but are higher than optimal. For these individuals, early intervention through lifestyle modifications can be crucial in preventing the progression to more severe lipid abnormalities and reducing the risk of cardiovascular diseases.

Lifestyle modifications encompass a variety of strategies, including increased physical activity, weight management, smoking cessation, stress reduction, reduction of salt in the diet, eating more fruit and vegetables, and avoiding harmful use of alcohol [8]. Among these, dietary changes stand out as a particularly impactful and accessible method for many individuals. Several dietary interventions have shown promise in modulating blood lipid profiles [9]. For instance, oats contain β-glucan, a soluble fiber demonstrated to reduce low-density lipoprotein (LDL) cholesterol levels by forming a gel-like substance in the gut that binds to cholesterol and reduces its absorption [10]. Similarly, docosahexaenoic acid (DHA), an omega-3 fatty acid found in fish oil, has been shown to lower triglycerides (TG) [11] and may also reduce the risk of heart disease by decreasing inflammation and improving endothelial function [12].

**Figure 1:**
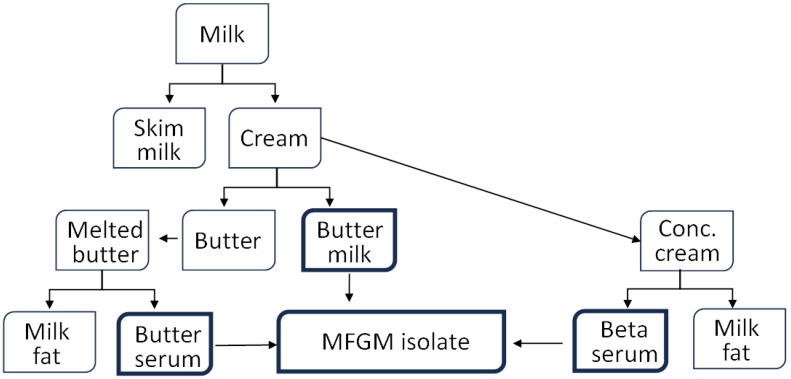
Isolation procedures of milk fat globule membrane (MFGM) from milk.

Dairy products have long been a staple in human diets, offering a rich source of essential nutrients, such as calcium, protein, and vitamins. Within the realm of dairy, milk fat globule membrane (MFGM) has garnered attention for its potential health benefits [13]. MFGM is a complex structure composed of lipids and proteins that surround fat droplets in milk. Emerging research suggests that MFGM may play a beneficial role in modulating blood lipid profiles, thereby contributing to improved metabolic health [14,15,16]. The process of obtaining MFGM involves the separation of milk fat from skim milk through centrifugation, followed by further processing to isolate the membrane components (Figure 1). Further details of the individual isolations are detailed in the individual references and the general isolation operations are illustrated by Fontecha et al. [17]. There are various strategies used in isolating a MFGM fraction, though the MFGM composition is similar. MFGM contains phospholipids (PLs) and sphingolipids (SLs), which are the main polar lipids in milk. PLs include sphingomyelin (SM), phosphatidylethanolamine (PE), phosphatidylserine (PS), phosphatidylcholine (PC), and phosphatidylinositol (PI). SM is the primary SL, with other SLs, such as glucosylceramide (GluCer), lactosylceramide (LacCer), and gangliosides, also present. PLs and SLs in MFGM affect lipid metabolism, gut microbiota, and inflammation, suggesting potential health benefits, particularly when comparing full-fat and lower-fat dairy products [13]. In the Western diet, daily intake is about 2–8 g for PLs [18] and 50–400 mg for SLs [19,20].

Despite extensive research, there has yet to be a meta-analysis specifically exploring the effect of bovine-sourced MFGM PL on serum lipid profiles in adults. Previous meta-analyses have shown that higher dairy intake is associated with a lower risk of metabolic syndrome in cohort studies (relative risk (RR) = 0.86; 95% CI: 0.79–0.92) and in cross-sectional and case-control studies (odds ratio (OR) = 0.83; 95% CI: 0.73–0.94). Additionally, a dose–response analysis of prospective studies suggested a 6% lower risk of metabolic syndrome with each one-serving increment of dairy consumption (RR = 0.94; 95% CI: 0.90–0.98). However, these studies did not consider the fat content of the dairy products used [21]. Since the fat in dairy is unique and may have lipid-profile-enhancing properties, it is crucial to specifically explore the effect of MFGM on lipid profiles.

This meta-analysis aims to investigate the effects of MFGM phospholipids on lipid profiles in healthy adults. By examining the collective findings of various studies, we seek to elucidate the role of MFGM on blood lipid profiles and its viability as a natural alternative or adjunct to pharmacological treatments.

## 2. Materials and Methods

### 2.1. Searching and Selection Processes

To identify all relevant studies published up to March 2024, a comprehensive literature search was conducted across various electronic databases, including PubMed, Scopus, Web of Science, the Cochrane Library, Google Scholar, ACS Publications, Academic Search Index, BMJ Journals, BNP Media, and others. The search strategy incorporated keywords and Medical Subject Heading (MeSH) terms, such as ‘milk fat globule membrane’, ‘MFGM’, ‘cholesterol’, ‘LDL’, ‘HDL’, ‘triglycerides’, ‘adults’, and ‘randomized controlled trial’, combined with relevant Boolean operators. Only English-language publications were included. Additionally, a manual examination of the reference lists of identified articles and reviews was performed to ensure the comprehensiveness of the search.

The PICO parameters were defined as follows:

- Populations: Adult human participants aged 20 years and older. No restrictions were based on ethnicity, sex, or baseline health status.
- Intervention: MFGM derived from bovine dairy products, such as cream or buttermilk. Studies involving other forms of dairy or non-dairy polar lipids were excluded.
- Comparator: A control group that received either a placebo or no supplementation. Studies without a clear control group or matched polar lipid amount were excluded.
- Outcome: Changes in blood lipid levels, specifically, total cholesterol (TC), LDL, HDL, and TG. Studies that did not measure at least one of these outcomes were excluded.

### 2.2. Inclusion and Exclusion Criteria

Trials were included if they met the following criteria: (i) the study design was limited to randomized controlled trials (RCTs), which could be either parallel-group or crossover design, (ii) participants were adults aged 20 years or older, (iii) the intervention group received MFGM phospholipids, and (iv) the outcomes included at least three of the following: TC, TG, HDL, and LDL. Studies that included any of these outcomes were considered for inclusion. The exclusion criteria were as follows: (i) randomized trials conducted in special populations, such as pregnant women, (ii) trials that lacked a control group, (iii) interventions that included other supplements of no interest, (iv) acute postprandial studies, and (v) animal or cell experiments, review conference papers, and literature with unavailable or unconvertible data.

The risk of bias for each trial was assessed using the Cochrane criteria, evaluating the following domains: (i) randomization, (ii) allocation concealment, (iii) blinding of participants and personnel, (iv) blinding of outcome assessors, (v) selective outcome reporting, (vi) incomplete outcome data, and (vii) other potential sources of bias. Trials were rated as (i) low risk of bias, (ii) some concerns—unclear risk of bias, (iii) high risk of bias, or (iv) no information. A trial was categorized as having a low risk of bias overall if all domains were rated as probably low risk of bias or low risk of bias. Conversely, a trial was categorized as having a high risk of bias overall if one or more domains were rated as probably high risk of bias or high risk of bias. Two investigators (A.P.K. and M.F) independently evaluated the potential risks of bias and resolved any discrepancies through discussion.

### 2.3. Data Extraction and Quality Appraisal

Two investigators (A.P.K and S.J.S.) independently assessed the titles and abstracts of the identified studies. For studies that potentially met the inclusion criteria, full texts were obtained. Any disagreements between the investigators regarding study eligibility were resolved through discussion or by consulting a third investigator. Data extraction was also performed independently by two investigators using a standardized form. The extracted information included study characteristics (author, year of publication, sample size, and duration), participant demographics, details of the MFGM intervention and control conditions, and lipid profile outcomes. Values reported in mg/dL for TC, LDL, and HDL were converted to mmol/L by dividing them by 38.67, while values for TG reported in mg/dL were divided by 88.57 to convert them to mmol/L.

### 2.4. Statistical Analysis

The meta-analysis was performed using Comprehensive Meta-Analysis (CMA) software v4.0. A random-effects model was employed to account for heterogeneity, assuming the studies represent a random sample from a broader population. Standardized mean differences (SMDs) and 95% confidence intervals (CIs) were calculated for each lipid outcome. Heterogeneity was assessed using the I² statistic. Publication bias was evaluated through funnel plots and Egger’s test, with additional sensitivity analyses conducted to examine the robustness of the findings. All analyses adhered to the guidelines for conducting and reporting meta-analyses.

## 3. Results

### 3.1. Results of the Search and Study Characteristics

**Figure 2:**
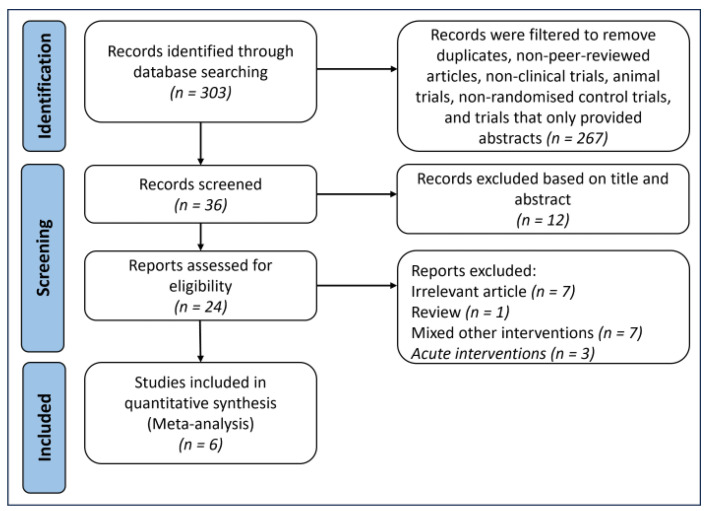
Study selection process based on PRISMA guidelines.

The search strategy yielded 303 articles from various databases. After removing duplicates and non-peer-reviewed studies, 36 studies remained. Screening based on titles and abstracts led to the exclusion of 12 studies, which included animal experiments, non-randomized controlled trials (non-RCTs), reviews, and studies using non-bovine-sourced MFGM or milk as the sole source of MFGM, leaving 24 articles. These 24 articles underwent a full-text review, resulting in the exclusion of 18 articles due to irrelevance (*n* = 7), mixed interventions (*n* = 7), and acute interventions (*n* = 3). A flowchart of the selection process is presented in Figure 2.

The details of the included studies are summarized in Table 1. A total of 464 adults participated in studies where MFGM phospholipids served as the primary dietary intervention. The participants were a mixture of men and women, with 115 being overweight. The studies were published between 1981 and 2020. Among the six studies, one was a crossover trial [14], while the remaining five were parallel controlled trials [15,22,23,24,25]. Additionally, one study included two active arms with different doses [15]. The intervention durations ranged from 3 to 16 weeks, with 3 studies lasting 4 weeks. The sources of MFGM phospholipids varied, including cream-based, buttermilk-based, and butter-serum- and beta-serum-derived types (Figure 1). Consequently, the doses tested varied widely. Most studies reported the phospholipid doses administered to participants, which ranged from 19.5 mg/day to 5 g/day; however, one study only reported the SM content, and another did not report any information. Only one study did not specify the phospholipid amount added to the diet. The choice of control also varied, with some studies using skim milk, whole milk, or rice powder. All studies reported total TC, TG, and HDL levels, and all but one study [22] reported LDL levels. All studies were conducted in adults, with a mean participant age above 40 years.

### 3.2. Risk of Bias Assessment

**Figure 3:**
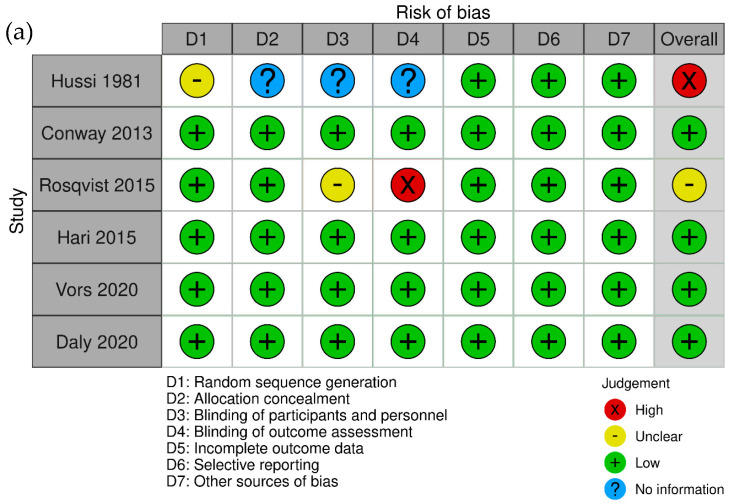

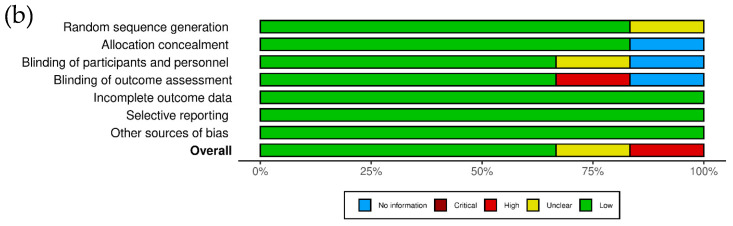
Assessment of bias risk in included studies: (**a**) bias risk summary—bias risk was classified as low (+), unclear (-), high (x), or no information (?) [14,15,22,23,24,25], and (**b**) bias risk graph—reviewing authors’ judgments about the bias risk of each item, shown as percentages across all included studies. Images were generated using the Robvis tool [26].

Overall, four trials were rated as low risk, one as high risk, and one as unclear. One trial was categorized as having an unclear risk of bias, while five trials were classified as having a low risk of bias in random sequence generation. Regarding allocation concealment, five trials were rated as low risk, and one trial lacked sufficient information to determine the risk of bias. Four trials were evaluated as having a low risk in the domain of blinding of participants, personnel, and outcome assessment, whereas two were deemed unclear (Rosqvist et al.’s [23] trial was single-blinded). All trials were evaluated as having a low risk of bias in the domains of incomplete data, selective reporting, and other sources of bias. Specifically, four trials were rated as low risk overall (Figure 3).

### 3.3. Total Cholesterol

**Figure 4:**
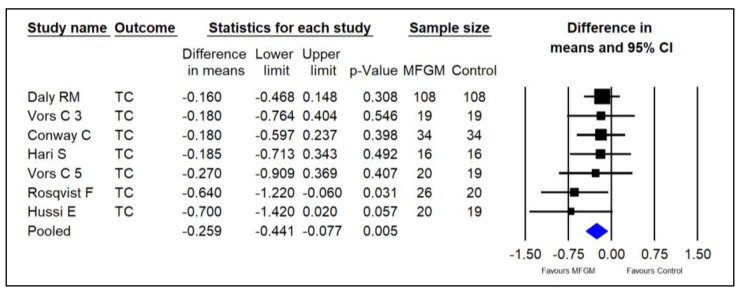
Effect of MFGM phospholipid supplementation on TC (mmol/L) in adults. In Vors et al.’s study [15], 3 and 5 refer to the experimental groups consuming 3 g and 5 g of milk polar lipids/day, respectively.

Six studies investigated the effect of supplementing MFGM phospholipids on serum TC in adults [14,15,22,23,24,25]. The pooled results indicated that MFGM PL may significantly decrease TC in healthy adults (SMDs = −0.259; 95% CI: −0.441~−0.077; *p* = 0.005; I^2^ = 0%; Figure 4).

### 3.4. Low-Density Lipoprotein Cholesterol

**Figure 5:**
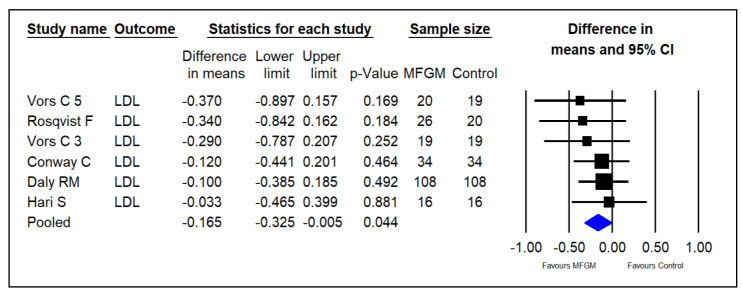
Effect of MFGM phospholipid supplementation on LDL (mmol/L) in adults. In Vors et al.’s study [15], 3 and 5 refer to the experimental groups consuming 3 g and 5 g of milk polar lipids/day, respectively.

Five studies investigated the effect of supplementing MFGM phospholipids on serum LDL in adults [14,15,23,24,25]. The pooled results indicated that MFGM PL may significantly decrease LDL in healthy adults (SMDs = −0.165; 95% CI: −0.325~−0.005; *p* = 0.044; I^2^ = 0%; Figure 5).

### 3.5. High-Density Lipoprotein Cholesterol

**Figure 6:**
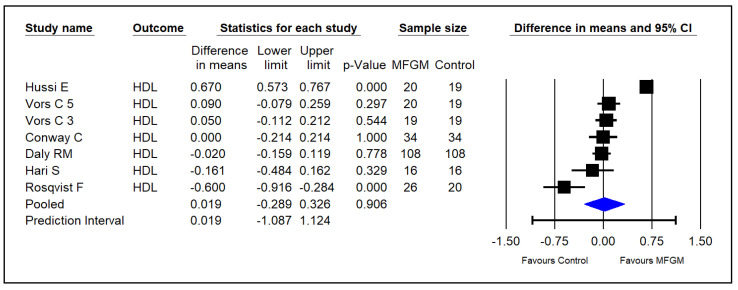
Effect of MFGM phospholipid supplementation on HDL (mmol/L) in adults. In Vors et al.’s study [15], 3 and 5 refer to the experimental groups consuming 3 g and 5 g of milk polar lipids/day, respectively.

Six studies investigated the effect of supplementing MFGM phospholipids on serum HDL in adults [14,15,22,23,24,25]. The pooled results indicated that MFGM did not significantly increase HDL in healthy adults (SMDs = 0.019; 95% CI: −0.289~0.326; *p* = 0.906; I^2^ = 95.5%; Figure 6).

### 3.6. Triglycerides

**Figure 7:**
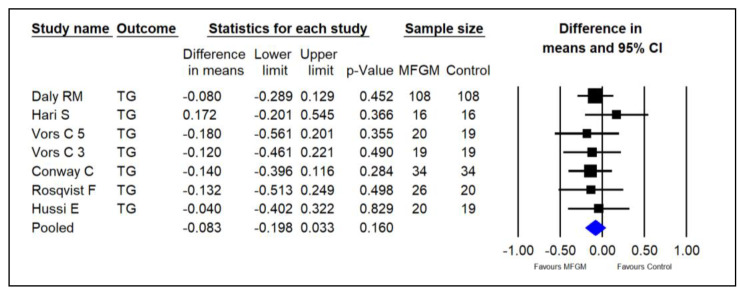
Effect of MFGM phospholipid supplementation on TG (mmol/L) in adults. In Vors et al.’s study [15], 3 and 5 refer to the experimental groups consuming 3 g and 5 g of milk polar lipids/day, respectively.

Six studies investigated the effect of supplementing MFGM phospholipids on serum TG in adults [14,15,22,23,25,26]. The pooled results indicated that MFGM did not significantly decrease TG in healthy adults (SMDs = −0.083; 95% CI: −0.198~0.033; *p* = 0.160; I^2^ = 0%; Figure 7).

### 3.7. Overall—Combined Analysis

**Figure 8:**
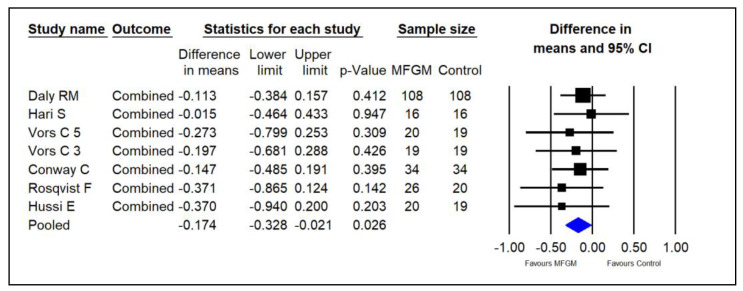
Effect of MFGM phospholipid supplementation on overall lipid concentrations (TC, LDL, and TG, in mmol/L) in adults. In Vors et al.’s study [15], 3 and 5 refer to the experimental groups consuming 3 g and 5 g of milk polar lipids/day, respectively.

An overall effect of supplementing MFGM phospholipids on serum lipids (TC, LDL, and TG) in adults was conducted to assess the effect on these three lipid markers irrespective of where they came from [14,15,22,23,24,25]. The pooled results suggested that MFGM may significantly decrease overall lipid levels in adults (SMDs = −0.174; 95% CI: −0.328~−0.021; *p* = 0.026; I^2^ = 0%; Figure 8).

### 3.8. Publication Bias

**Figure 9:**
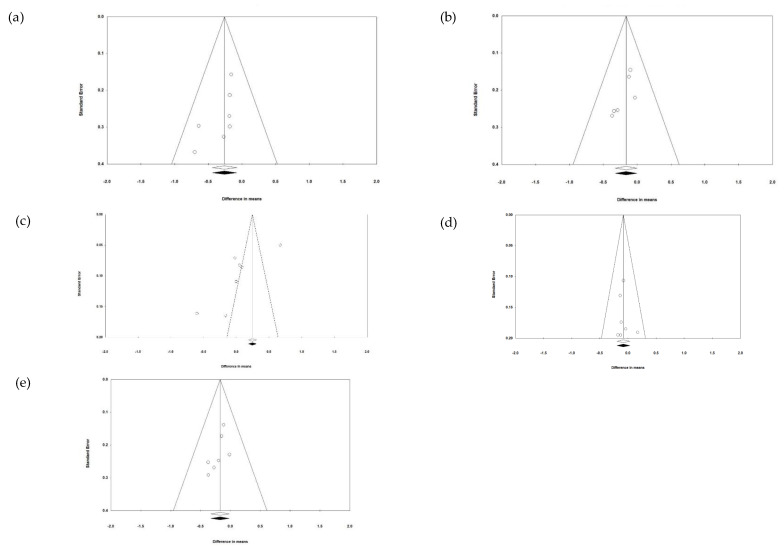
Funnel plots to evaluate publication bias and the effect of PL (MFGM) supplementation for (**a**) TC, (**b**) LDL, (**c**) HDL, (**d**) TG, and (**e**) combined in participating in the response to MFGM.

Funnel plots were utilized to evaluate publication bias (Figure 9). Upon visual inspection, the funnel plots for TC, LDL, and the combined analysis appeared symmetrical, with even scattering of the data, indicating no publication bias. These are the only outcomes for which the meta-analysis was significant. Conversely, the funnel plot for HDL exhibited marked asymmetry (Figure 9c), suggesting a high level of publication bias, likely attributable to the limited number of studies for this outcome. This observation was corroborated by Egger’s test for HDL, which yielded a t-value of 3.301 (one-tailed *p* = 0.0107, two-tailed *p* = 0.0215). The intercept was −9.754 (95% CI: −17.350 to −2.158), indicating significant publication bias. Additionally, Egger’s test indicated a borderline presence of publication bias for TC (t = 1.968, one-tailed *p* = 0.0531, intercept = −1.703, 95% CI: −3.927 to 0.521), LDL (t = 2.316, one-tailed *p* = 0.041, intercept = −1.778, 95% CI: −3.910 to 0.354), and the combined analysis (TC, TG, and LDL; t = 1.882, one-tailed *p* = 0.059, intercept = −1.277, 95% CI: −3.021 to 0.467). Egger’s test indicated no significant publication bias for TG (t = 0.421, one-tailed *p* = 0.346, intercept = 0.457, 95% CI: −2.335 to 3.249). Since the meta-analysis was not significant for HDL and TG, the publication bias for these outcomes was not as relevant.

## 4. Discussion

In this meta-analysis of 6 trials comprising 464 adults, we found that MFGM phospholipids were associated with reductions in certain blood lipid measures. Specifically, there was a significant change in serum TC and LDL levels following supplementation of MFGM phospholipids in healthy adults. These findings provide evidence that MFGM supplementation may have a small but statistically significant effect on lowering blood lipid levels, suggesting that MFGM could be a beneficial dietary intervention for managing dyslipidemia and reducing cardiovascular risk.

This meta-analysis contributes to the evolving narrative around dairy products and their impact on health, particularly in the realm of blood lipid management. As mentioned briefly above, higher dairy intake is associated with a lower risk of metabolic syndrome [21]. In a recent review, dairy fat intake was reported to have neutral to beneficial cardiometabolic activity [16]. Furthermore, results of meta-analyses have observed no detrimental effects of high-dairy (irrespective of fat content), low-fat dairy, or full-fat dairy diets compared with control groups (low in dairy) on LDL, HDL, and TG, though there is a suggestion that there is a positive effect on HDL (SMDs of 0.26 mmol/L) [27]. Extensive reviews of the cardiometabolic benefits of dairy [16] and MFGM on health have been published [13,28]. This meta-analysis is the first to specifically examine RCTs of MFGM phospholipids on blood lipids.

How MFGM may be providing benefits to blood lipids is not fully understood; however, animal studies provide some initial insights, demonstrating reductions in plasma LDL [29] and TC [30,31]. Beyond blood lipids, MFGM phospholipid supplementation in rodent studies has shown several additional benefits: preventing weight gain [32], modulating gut physiology (gut permeability, colonic crypt depth, increased goblet cells, and changes in the microbiome) [30,33,34], reducing inflammatory markers [33,34], improving liver conditions (reducing hepatic steatosis and hepatomegaly) [31,35,36], and increasing fecal cholesterol excretion [35]. Beyond animal studies, clinical trials included in this meta-analysis have also measured other parameters that may elucidate the mechanism. For example, both Rosqvist et al. [23] and Vors et al. [15] found reduced apoB:apoA-I ratios in the MFGM PL group, while Conway et al. [14] observed that buttermilk consumption was associated with changes in plasma β-sitosterol, another marker of cholesterol absorption. In another study, Conway et al. [37] observed a significant reduction in systolic and arterial blood pressure, as well as plasma levels of the angiotensin-converting enzyme (ACE) in response to buttermilk consumption. These findings illustrate the complex relationship between MFGM phospholipids and various aspects of metabolic health, highlighting their multifaceted impact on the body.

As described above, MFGM PL affects multiple aspects of lipid metabolism, ultimately impacting metabolic health. This is predominantly attributed to the PL content of MFGM. The mechanistic potential of PL having an impact on lipid metabolism is not novel. It is known that dietary PLs can inhibit intestinal lipid absorption when added to the diet in significant amounts by interfering with lipid mobilization from mixed micelles [18,38]. The different types of PL have also been shown to have differential effects. The presence of PC in taurocholate containing mixed micelles reduced the uptake of cholesterol by Caco-2 cells [39]. SM was shown to dose-dependently reduce the intestinal absorption of TC, TG, and fatty acids in rodents [30]. Products of SM digestion, such as ceramides and sphingosine, also inhibit cholesterol and fatty acid absorption [13]. Other milk polar lipids, such as PC, PE, and gangliosides, are also known to reduce the intestinal absorption of dietary lipids. When present in the bile, PC can facilitate intestinal absorption of dietary lipids and can inhibit lipid absorption when present in large amounts in the diet. Furthermore, studies have reported the impact of individual PL on serum lipids and lipid absorption in in vitro, rodent, and human studies [40,41,42,43,44,45]. These findings collectively illustrate that, while individual PL compounds may confer specific health benefits, dietary intake typically involves whole foods rather than isolated compounds. Dairy products, especially MFGM, being a rich source of various PL, offer a comprehensive array of these beneficial compounds, thereby enhancing their overall nutritional value.

There are various foods and supplements marketed for their blood-lipid-lowering benefits. For example, meta-analyses of β-glucan from oats have shown promising results. Xu and Sun [46] reported that β-glucan could potentially reduce TC by 0.27 mmol/L and LDL by 0.26 mmol/L, which is consistent with more recent findings by Yu et al. [47] showing reductions in TC by 0.24 mmol/L and LDL by 0.27 mmol/L. No significant effects were found on HDL and TG in these analyses. Similarly, fish oil or omega-3 supplements, which are marketed for their benefits on blood lipid profiles, have shown potential in reducing TG by 0.39 mmol/L in patients with metabolic syndrome, according to a meta-analysis [48]. Further, a meta-analysis by Zhang et al. [49] explored the differential impacts of DHA and EPA, both of which were shown to reduce TG levels. In this context, the results of the meta-analysis on MFGM indicated potential benefits on TC and LDL, suggesting that MFGM phospholipids could also play a role in lipid-lowering strategies. This provides consumers with additional options beyond oats and fish oils, potentially broadening the range of dietary interventions available for improving lipid profiles and reducing cardiovascular risk. In addition, the presence of MFGM may help explain the null or positive effects found for the full-fat dairy impact on cardiovascular disease risk factors [50].

This meta-analysis is not without its limitations. The current evidence is constrained by the small number of studies and the potential presence of heterogeneity, making it challenging to provide definitive recommendations and necessitating cautious interpretation. Also, the literature search was completed in March 2024 and may have missed any new research after this date. Additionally, the analysis examined the effects of MFGM PL interventions with a dose range spanning from 19.8 to 5000 mg. While this broad range allows for a comprehensive overview, it introduces significant heterogeneity, complicating the identification of an optimal dose and reducing the generalizability of the findings. Furthermore, the meta-analysis of MFGM and blood lipids revealed some publication bias for the significant outcomes, particularly for TC, LDL, and the combined analysis. This bias suggests that the observed effects may be somewhat overestimated due to the limited number of studies. Despite these limitations, the results still provide valuable insights into the potential benefits of MFGM on lipid profiles. To address the issue of publication bias and strengthen the evidence base, further research is needed. Specifically, larger and well-designed randomized controlled trials should be conducted and published, regardless of their outcomes. Additionally, future studies should include comprehensive reporting and consider dose–response assessments to determine the optimal dosage required to achieve positive benefits.

## 5. Conclusions

The evidence from this meta-analysis indicated that MFGM may have a positive or neutral effect on blood lipid levels. However, the extent of this effect appears to vary across different populations. Further research involving larger sample sizes and more diverse populations is necessary to confirm these findings and to better understand the potential variability in the impact of MFGM on blood lipid profiles.

## Figures and Tables

**Table 1:** Description of the studies included in this meta-analysis.

Author	Country	Age, Range (Mean or Median) y	Participants	Study Design	Intervention	Control Group	Intervention Duration	Outcomes
Conway et al. [14]	Canada	18–65 (49.4 ± 12.8 y)	34 men and women	Crossover	45 g skim milk + buttermilk powder mixed with water (187.5 mg phospholipids)	45 g skim milk powder mixed with water (34.6 mg phospholipids)	4 weeks	TC, TG, HDL, LDL
Vors et al. [15]	France	56–62 (49.4 ± 12.8 y)	58 overweight postmenopausal women (20 in 5 g experimental group, 19 in 3 g experimental group, 19 in control group)	Parallel	Full-fat cream cheese with 3000 mg or 5000 mg of phospholipids	Full-fat cream cheese with 0 mg phospholipids	4 weeks	TC, TG, HDL, LDL
Hussi et al. [22]	Finland	Not provided in text	39 healthy male prisoners (20 in experimental group, 19 in control group)	Parallel	2.0 L buttermilk	3 dL milk	3 weeks	TC, TG, HDL
Rosqvist et al. [23]	Sweden	20–70 y (median 58.5 y in control and 60.5 y in MFGM)	57 overweight men and women (26 in experimental group, 20 in control group)	Parallel	1 dL whipping cream (40% fat)/d, 1 dL fat-free milk (0.1% fat)/d, and 1 scone/d (19.8 mg phospholipids)	1 dL fat-free milk (0.1% fat)/d and 1 scone/d (1.3 mg phospholipids)	8 weeks	TC, TG, HDL, LDL
Hari et al. [24]	Japan	20–64 (42.0 ± 11.9 y)	32 healthy men and women (16 in experimental group, 16 in control group)	Parallel	6.5 g MFGM tablet (231 mg sphingomyelin)	6.5 g whole milk powder tablet (4 mg sphingomyelin)	4 weeks	TC, TG, HDL, LDL
Daly et al. [25]	Australia	45–65 (55 ± 5 y in MFGM, 56 ± 5 y in placebo)	244 healthy women (123 in experimental group, 121 in control group)	Parallel	60 g milk powder + MFGM mixed with water (400 mg phospholipids)	60 g rice powder mixed with water (0 mg phospholipids)	16 weeks	TC, TG, HDL, LDL

## Data Availability

The original contributions presented in the study are included in the article, further inquiries can be directed to the corresponding author.
